# The relationshipbetween anxiety and social outcomes in autistic children and adolescents: A meta-analysis

**DOI:** 10.1007/s10567-023-00450-7

**Published:** 2023-08-22

**Authors:** Dawn Adams, Kathryn Ambrose, Kate Simpson, Stephanie Malone, Nicole Dargue

**Affiliations:** 1 Autism Centre of Excellence, Griffith University, Messines Ridge Road, Mt Gravatt, QLD 4122 Brisbane, Australia; 2 Griffith Institute for Educational Research, Griffith University, Messines Ridge Road, Mt Gravatt, QLD 4122 Brisbane, Australia; 3 Autism CRC, Brisbane, Australia

**Keywords:** Autism, Youth, Anxious, Mental health, Social skills

## Abstract

Anxiety is one of the most frequently reported co-occurring conditions for autistic children and adolescents. The relationship between anxiety and social outcomes in autistic youth has been the focus of a range of studies, with mixed results. This meta-analysis aimed to identify the strength of the association between anxiety and a frequently researched social outcome (social competence) in autistic young people and whether that association is influenced by individual or research design factors. A previous preregistered systematic review was updated with a search of the same three databases (CINAHL, ERIC, and PsycINFO) as the original review. Through this, 20 studies with sufficient data on a neurotypically-defined measure of social competence and anxiety were identified. Results were synthesised using a mixed effects model. The meta-analysis on 2,321 participants (from 22 samples) highlighted wide heterogeneity in results. The findings show that anxiety has a significant, small negative impact on social competence (*d* = − 0.48; 95% *CI* = − 0.71, − 0.26), meaning that as scores on measures of anxiety increase, scores on measures of social competence decrease. This relationship between anxiety and social competence was moderated by age, becoming weaker as age increased. Whilst this is an important finding for supporting mental health and well-being of autistic young people, the large amount of variance left unexplained suggests that multiple factors, including the use of measures designed for neurotypical people and the potential impact of camouflaging on such measures, need to be considered in future designs.

Anxiety is one of the most common co-occurring conditions in autistic children and adults (Hollocks et al., 2019; Hossain et al., 2020). The last 15 years has seen a large increase in the volume of research in this area (Vasa et al., 2018), with studies reporting upon the prevalence of and presentation of anxiety from the preschool years (Keen et al., 2019) into old age (Uljarević et al., 2020). Meta-analyses have shown that autistic children and young people experience elevated levels of anxiety compared to non-autistic youth (van Steensel & Heeman, 2017) and that levels of anxiety are higher in autistic individuals without an intellectual disability than in those with a co-occurring intellectual disability (Hollocks et al., 2019; Mingins et al., 2021).

With the pervasiveness and chronicity of anxiety in autistic people now well established, research has begun to document the impact that anxiety is having on autistic people’s lives. Individually, studies have shown that autistic people with elevated anxiety have a poorer quality of life (Adams et al., 2020; Lin & Huang, 2019), experience higher levels of anger and behaviours of concern (Kerns et al., 2015; Townsend et al., 2022), and participate less in home and community activities (Ambrose et al., 2022). Qualitative studies have also documented how anxiety can impact upon many facets of autistic people’s lives (Robertson et al., 2018) by limiting options and making even enjoyable activities challenging (Ong et al., 2023).

In an attempt to identify the impact of anxiety on social and academic outcomes of autistic youth, Ambrose et al. (2021) conducted a systematic review of the research in the area. They identified 50 studies; 47 reported upon anxiety and social outcomes and just three reported on anxiety and scores on academic measures. Using the same categorisation of social outcomes as de Lijster et al. (2018), who conducted the same systematic review but in neurotypical children, Ambrose et al. identified five studies that explored the relationship between anxiety and social relationships, five that explored the relationship between anxiety and victimisation and social acceptance, and 40 studies that explored the relationship between anxiety and social competence. Social competence is a broad term referring to (neurotypically-defined) skills used to establish social relations with peers and adults, to form friendships, and to understand the needs of others (Hoffman et al., 2015). It includes a range of cognitive skills and processes such as social motivation, social inferencing, empathy, social knowledge, verbal conversation skills, nonverbal communication skills, and emotional regulation (Scheerer et al., 2020). It is important to acknowledge that the concept of social competence is defined by neurotypical norms rather than by autistic people themselves. Social competence is complex and multifaceted; no single behaviour is sufficient to measure social competence, and social competence in one situation may not necessarily be transferable to other situations (Hukkelberg et al., 2019). The broad description leads to a variety of measures used to measure social competence, including measures of social skills, abilities, or functioning, emotional understanding of social contexts, and/or measures of social difficulties.

The role of social competence in neurotypical development has been explored in depth, with consistent findings showing that higher social competence is linked to better self-esteem, while lower social competence is linked to higher rates of internalising disorders such as anxiety (Bornstein et al., 2010; Joy, 2016). This inverse relationship between social competence and internalising symptoms has been repeatedly reported in neurotypical children as young as 3–6 years old (Huber et al., 2019). This suggests that social competence may have an important, and potentially reciprocal, role in supporting the psychological well-being and in minimising mental health challenges of neurotypical children and adolescents. However, the findings of the Ambrose et al. (2021) review showed a less consistent relationship between anxiety and social competence in autistic young people. Of the 40 studies reporting on anxiety and social competence in their review, 29 reported correlational findings. Of these, 17 reported a significant, negative relationship, but seven reported no relationship. Further examples of the variability of the results were shown through the nine studies that compared social competence in autistic children in higher and lower anxiety groups; three reported poorer social competence across all social measures in children with higher anxiety, two showed mixed results, and four showed no difference between the groups.

As mentioned previously, social competence is a multifaceted construct and the relationship between social competence and anxiety in autistic individuals may differ depending on how it is measured. The broad definition and therefore broad range of ways to measure social competence may in part, explain the inconsistent findings with regards to the relationship between anxiety and social competence in autistic young people. However, given such inconsistency is not noted in neurotypical youth, there may be a need to consider other factors which are more pertinent to autistic young people. One possible these may be the differing presentation of anxiety in relation to the person’s sex/gender (e.g., Ambrose et al., 2020), or the fact that autistic females are more likely to show camouflaging and masking which in turn changes the observable social behaviours that are often the focus of questionnaires (O’Loghlen & Lang, 2023). Inconsistent findings on the relationship between social competence and anxiety may also be influenced by individual characteristics, such age or level of cognitive ability, both of which have been shown to impact scores on anxiety measures in previous meta-analyses (Spackman et al., 2022; van Steensel & Freeman, 2017). Finally, social competence may also be influenced by the social partner and the person providing the ratings, explained by the double empathy problem theory (Milton, 2012). This theory suggests a two-way problem with perspective taking which impacts upon social interactions and experiences, that is, non-autistic people can have challenges understanding autistic people just as much as autistic people can have challenges understanding non-autistic people.

Given the variability in the findings of studies reporting on anxiety and social competence in autistic young people, a meta-analysis is now warranted to build upon the descriptive synthesis of the extant literature by Ambrose et al. (2021) and examine the overall direction and strength of the relationship (if any) between anxiety and social competence. Having a clear picture of how anxiety and social competence are associated with each other is important as many autistic people desire friendships (Sosnowy et al., 2019). If these results suggest that anxiety is impacting upon social interactions, it provides a potential way to support social relationships and friendships through reducing anxiety. The aim of this study was therefore to use a meta-analytic technique to aggregate data from across multiple studies identified through a systematic review to address the following questions about the relationship between anxiety and social competence in autistic children:

1. What is the strength of the association between anxiety and social competence in autistic young people?
2. Do individual or research design factors (i.e., participant sex/gender, age, presence of ID, study quality, and whether a broad or specific measure of social competence was used) moderate this relationship?

Due to the variability in the research findings reported in Ambrose et al.’s (2021) systematic review, no directional hypotheses were made.

## Ethics

The present meta-analysis used pre-existing anonymised data from published manuscripts and therefore was exempt from approval by the Ethics Committee of the authors’ institution.

## Method

As the need for this meta-analysis is based upon the findings of the Ambrose et al. (2021) systematic review, their original procedure (as documented in Prospero, registration number CRD42020142137) was followed and refined in the final stage (data extraction) to focus specifically on studies that report on anxiety and social competence. The reporting of this meta-analysis is in accordance with the Preferred Reporting Items for Systematic Review and Meta-Analysis Protocols (PRISMA-P) statement.

### Eligibility Criteria

Inclusion criteria for this meta-analysis were the same as for the Ambrose et al. (2021) systematic review, with the addition that included studies had to contain sufficient data on the relationship between anxiety and social competence for a meta-analysis. That is, (1) Participants had a diagnosis of autism provided by clinical report or a standardised measure; (2) Participants were under 18 years of age; (3) Anxiety was reported using a standardised, quantitative measure of anxiety or the proportion of participants with a community diagnosis of anxiety; (4) The social competence outcome was reported using standardised, quantitative measures; (5) Analysis involving the measure of anxiety and social competence outcome (for descriptive studies) or a pre-post intervention analysis of anxiety and social competence (for intervention studies) either reported an effect size, or sufficient data were reported for Cohen’s *d* to be calculated; (6) Studies reported original research published in English in a peer-reviewed journal, in or after 1994.

### Search Strategy

In October 2022, an update search was undertaken with the same electronic databases and terms that were last searched by Ambrose et al. in August 2021 (CINAHL, ERIC, and PsycINFO). The search terms were: autism (autis*, ASD, Asperger*, pervasive developmental disorder, PDD*), anxiety (anxiet*, phobia, fear, comorbid disorder, panic, comorbidity, psych*, agoraphobia, obsess*, internali*, mental health,) social outcomes [(social*, interpersonal or peer) AND (functioning, interaction, skill, impair*, participation, behavio* outcome*, competen*, performance problem*) OR bully* OR friend*)] OR academic outcomes [(academic*, school, student, education*) AND (achievement, behavio*, class, competen*, grade, failure, functioning, impair*, performance, problem, refusal, outcome, retention, stress, skill*, underachievement, participation)]. Using covidence, screening of title and abstracts was undertaken by a single reviewer with a second independent reviewer screening a randomly selected sample of 20%, resulting in 100% agreement. Studies which were deemed potentially relevant at the title and abstract stage went through to full-text review. All studies which went through to this stage were reviewed by two independent reviewers, resulting in 100% agreement. No studies met the full inclusion criteria for the meta-analysis from this updated search. Therefore, the meta-analysis was based upon studies listed in Ambrose et al. (2021) as reporting on social competence and anxiety to provide the dataset of included studies for this meta-analysis.

### Data Extraction and Study Quality

For each paper, information extracted included study details (including geographical location of study), sample characteristics (sex, prevalence of ID, age), and study design (measures used). Data from the measure of anxiety and social competence used in the study, including effect sizes, were also extracted. Data extraction was undertaken for all included papers independently by two of the authors; 100% agreement was reached. Where an included study reported sufficient data to calculate an effect size on multiple subscales from a measure or on multiple scales of social competence, the a priori decision was taken to extract data for the meta-analysis from the more precise subscale (e.g., the anxiety subscale in preference to the internalising subscale) or scale. Measures of *social competence* were classified as specific if they were focussed solely on social competence (e.g. Multidimensional Social Competence Scale) or broad if they measured social competence as part of broader measure of social skills or styles (e.g. Vineland Adaptive Behaviour Scales, Socialisation score).

Included studies were evaluated for methodological quality using the STROBE checklist (von Elm et al., 2007). This 22-item checklist provides a broad assessment of study quality based on rationale, methods, analysis, reporting, and risk of bias. It can be used with a range of study designs. Following the same process as Ambrose et al. (2021), and as per Fortin et al. (2012), one point was awarded for each item, meaning that the maximum score was 22. Studies with a score of less than 11 (i.e., 50%) were excluded.

### Meta-Analytic Procedure

Data were analysed using Stata v. 17 (StataCorp, 2021). The effect size (Cohen’s *d*) was interpreted using Cohen’s (1988) guidelines, where *d* = 0.2 reflects a small effect size, *d* = 0.5 a medium effect size, and *d* = 0.8 a large effect size. If Cohen’s *d* was not reported in the article, it was calculated based on the data provided (if possible), following the processes and hierarchy stated in Adams et al., (under review): if standard errors were given, these were transformed into standard deviations and entered into the abovementioned formula. If this was not possible, associated *F*- or *t*-values were used in conjunction with applicable means to calculate pooled standard deviation (Johnson & Eagly, 2014). If that was not possible, the associated *p*-value was used to calculate the *t*-value, which was then used to obtain the pooled standard deviation (Johnson & Eagly, 2014). Finally, Hedges’ (1981) adjustment was applied to calculate unbiased estimates of Cohen’s *d*. Unbiased estimates of Cohen’s *d*, on average, neither over- nor under-estimate effect size, so the use of such an adjustment is particularly important when conducting a meta-analysis incorporating small sample sizes due to the associated risk of overestimating effect size (Cumming et al., 2012). Estimates of standard error around unbiased Cohen’s *d* were calculated using Lipsey and Wilson’s (2001) formula recommended for within-subjects designs.

## Results

### Search Results and Characteristics of the Studies

**Fig. 1:**
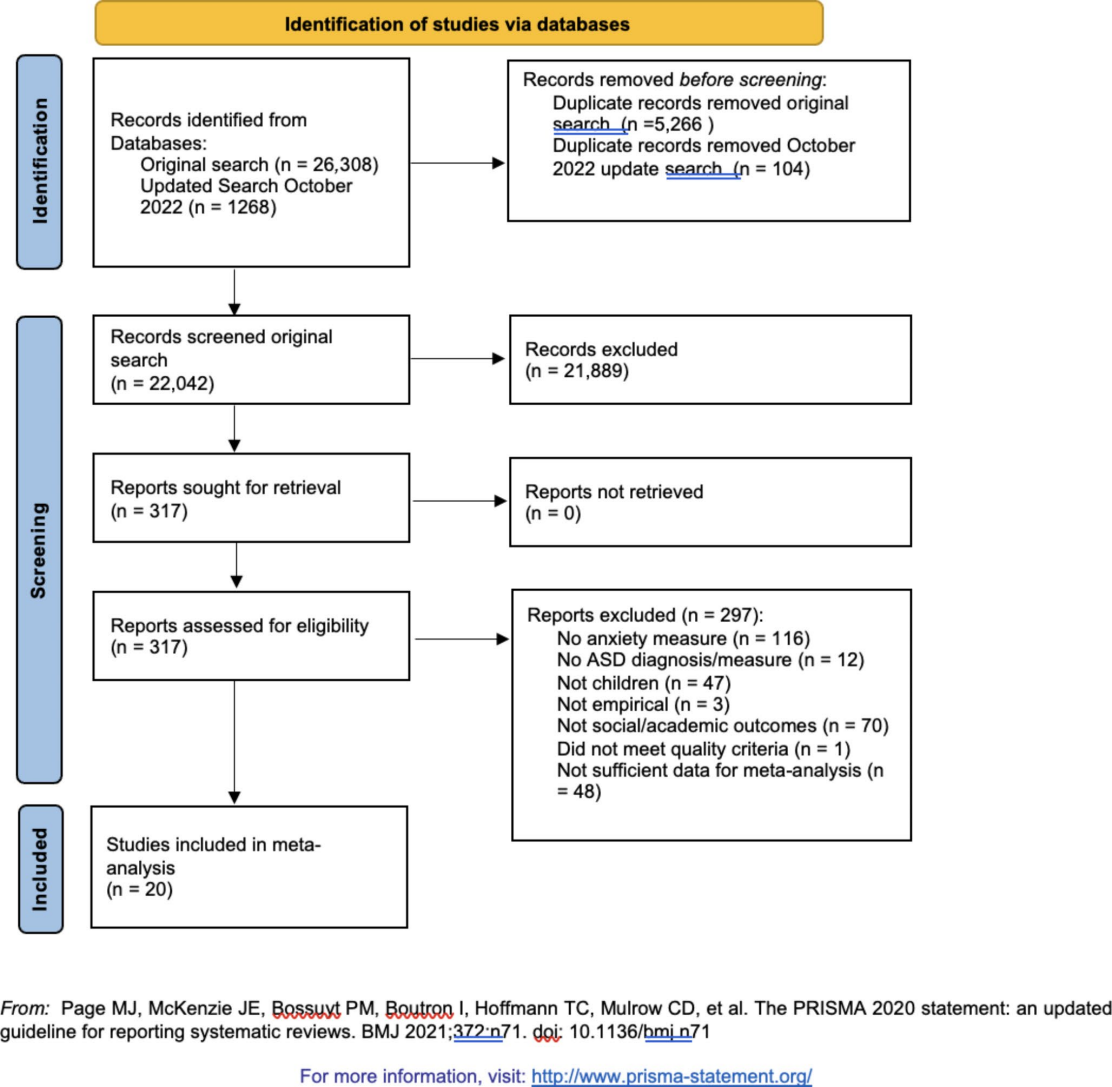
*PRISMA Diagram*

The process of the study selection from the original and updated search is provided in Fig. 1. The update search resulted in 1,268 new electronic records being identified (see Fig. 1). Following removal of duplicates, the titles and abstracts of 1,164 reports were assessed against inclusion criteria by the same primary rater as the original search, who had achieved inter-rater agreement with the lead author of 97.4% agreement (Kappa = 0.89). There were 107 articles in the update search where inclusion or exclusion could not be determined by the title and abstract alone. The full text of these 107, plus the 210 articles from the original search, were assessed against the meta-analysis inclusion criteria stated above. This resulted in a total of 20 studies (22 samples) that had sufficient data to be included in the meta-analysis. Table 1 summarises the included studies.

**Table 1:** *Studies Included in Meta-Analysis (Ordered Alphabetically)*

Study	*N* autistic	Age in years Mean (SD), range	% male	IQ	Study design	STROBE rating		Anxiety measure (data source)	Social outcome measure/s (data source)
Alkire et al. (2021; Parent, Child)	49	11.48 (2.11) 7.11–14.86	98	FSIQ > 80	D	20	SCARED social anxiety subscale (C, P)		ADOS CSS Social affect subscale (C)
Bitsika and Sharpley (2017; Child)	90	8.86 (1.94) 6–12	100	FSIQ 96.5 (12.3)	D	18	CASI- GAD subscale (P)		SRS Total score (P)
Bitsika and Sharpley (2017; Adolescent)	60	14.65 (1.43) 13–18	100	FSIQ 95.7 (11.9)	D	18	CASI- GAD subscale (P)		SRS Total score (P)
Corbett et al. (2017)	30	INT 11.27 (2.51) WLC 10.74 (1.89) 8–14	80	FSIQ > 70	INT	19	STAI-Trait (C)		PIP group play observation (C)
Duvekot et al. (2018)	79	8.4 (2.3) 4–13	81.5	FSIQ 50–141	D, L	19	CBCL anxiety DSM-orientated scale (P)		SRS SCI domain score (P)
Factor et al. (2016)	44	6.91 (3.64) 2–17	80	85.14 16–125	D	18	CBCL anxiety DSM-orientated scale (P)		SRS SM subscale (P)
Factor et al. (2017)	57	7.25 (3.85) 3–17	82.5	NR	D	18	CBCL anxiety DSM-orientated scale (P)		SRS Total (P)
Hallett et al. (2013)	415	8.47 (2.87) 4–17	85	NR	D	19	CASI anxiety scale (P)		VABS socialisation (P)
Study	*N* autistic	Age in years Mean (SD), range	% male	IQ	Study design	STROBE rating	Anxiety measure (data source)		Social outcome measure/s (data source)
Hill et al. (2014)	102	NR 6–13	82.4	NR	D	16	BASC anxiety subscale (P)		BASC social skills subscale (P)
Hollocks et al. (2014)	90	15.5 (0.47) 14.7–16.8	91.1	FSIQ 50–119	D	18	Anxiety scale derived from emotional items on PONS and SDQ (P)		Direct assessment (C)
Johnston and Iarocci (2017)	67	9.82 (2.11) 6–14	85	FSIQ > 70	D	14	BASC-2 anxiety subscale (P)		Multidimensional Social Competence Scale (P)
Kerns et al. (2015)	59	10.56 (2.75) 7–17	78	FSIQ > 60	D	18	ADIS-C/P principal anxiety, clinical severity rating (P)		SSRS assertiveness subscale (P)
McVey et al. (2018)	113	13.47 (1.41) 11–16	87.6	FSIQ 68–144	D	20	CBCL anxiety DSM-orientated scale (P)		SRS SCI score subscales (P)
Meyer et al. (2006)	31	10.1 (1.9) 7:9–13:9	83.9	ID excluded	E	14	SASC-R FNE subscale (C)		Social Competence Inventory (P)
Niditch et al. (2012)	231	5.0 (2.0) 2–9	84	NR	D	17	BASC-2 anxiety subscale (P)		BASC-2 social skills subscale (P)
Ratcliffe et al. (2015)	292	9.40 (1.39) 6–13	89	FSIQ > 50	D	18	DBC anxiety subscale (P)		SSIS-RS total score (combined P and T)
Sofronoff et al. (2017)	38	9.56 (NR) 7:11–12	87.8	FSIQ > 85	INT	18	SCAS (P)		Social Skills Questionnaire (P)
Study	*N* autistic	Age in years Mean (SD), range	% male	IQ	Study design	STROBE rating	Anxiety measure (data source)		Social outcome measure/s (data source)
Sukhodolsky et al. (2008)	171	8.2 (2.6) 5–17	84.2	MA > 18mths	D	18	CASI anxiety scale (P)		VABS socialisation (P)
Sukhodolsky et al. (2020)	180	4.70 (1.04) 3–7	88.1	FSIQ < 70	D	20	ECI anxiety subscale (P)		VABS socialisation (P)
Teh et al. (2017)	54	T1 10 (32.8) 5–17	88.9	NR	D, L	18	T1 SCAS total score (P)		T2 DBC-ASA S/C composite score (P)
White and Roberson-Nay (2009)	20	12.08 (1.78) 7–14	90	NR	D	15	CBCL anxiety DSM-orientated scale (P)		Social Competence Inventory (P)

*Note*: IQ: FSIQ = full-scale IQ; MA = mental age; NR = not reported; ID = intellectual disability

Study design or participant group: D = descriptive/non-experimental; E = experimental; INT = intervention WLC = Wait List Control. L Longitudinal

Data source: (P) = parent; (C) = child

Measures: ABC = Aberrant Behaviour Checklist; ADIS = Anxiety Disorders Interview Schedule; ADOS = Autism Diagnostic Observation Schedule; BASC-2 = Behavioural Assessment System for Children (2nd Edition); CASI = Childhood Anxiety Sensitivity Index; CBCL = Child Behaviour Checklist; DBC = Developmental Behaviour Checklist; ECI = Early Childhood Inventory; PIP = Peer Interaction Paradigm; SASC-R = Social Anxiety Scale for Children-Revised; SCARED = Screen for Child Anxiety Related Disorders; SCAS = Spence’s Children’s Anxiety Scale; SDQ = Strengths and Difficulties Questionnaire; SRS = Social Responsiveness Scale; SSIS = Social Skills Improvement System; SSRS = Social Skills Rating System; STAI = State Trait Anxiety Inventory; VABS = Vineland Adaptive Behaviour Scales.

### Meta-analysis

A random-effects model was used for all analyses given the significant heterogeneity identified in the literature surrounding the relationship between anxiety and social competence in autistic children (e.g., Ambrose et al., 2021; see here for dataset and analyses completed). In addition, Cochran’s test (DerSimonian & Laird, 1986) was used to identify heterogeneity within this specific sample. Cochran’s *Q* = 580.34, *p* < .001, suggesting that the included studies were heterogeneous. To determine the percentage of heterogeneity present between included studies, *I*^*2*^ was used such that *I*^*2*^ = 25% would indicate a small amount of heterogeneity, *I*^*2*^ = 50% would indicate a medium amount of heterogeneity, and *I*^*2*^ = 75% would indicate a large amount of heterogeneity (Higgins & Thompson, 2002; Higgins et al., 2003). A large amount of heterogeneity remained in this meta-analysis, with the meta-analysis explaining 4.21% of the total between-study variation (*I*^*2*^ = 95.79%). Such a finding was unsurprising given the different methods used across studies (e.g., different measures of social competence, different informants rating social competence, etc.).

#### The Relationship Between Anxiety and Social Outcomes

**Fig. 2:**
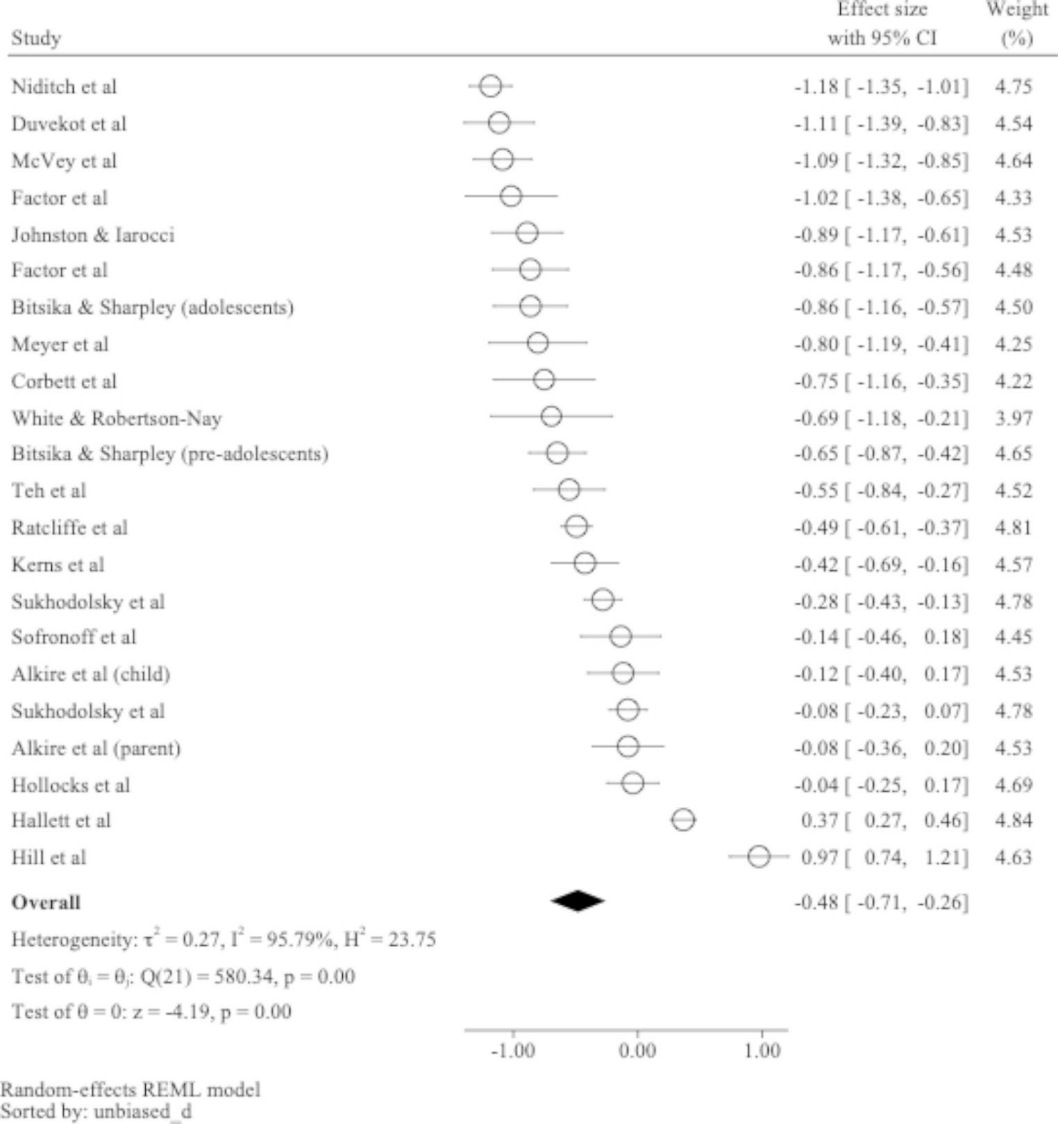
*Forest Plot of Effect Sizes*

A meta-analysis was undertaken on 22 independent samples from 20 articles identified in order to statistically determine whether there is any relationship between anxiety and social competence in autistic young people. Sample sizes of the studies included within this meta-analysis ranged from 20 to 415 participants (*M* = 105.50, *SD* = 97.92), with a total of 2,321 participants represented within the current meta-analysis overall. The calculated effect sizes ranged from − 1.18 to 0.97 (*M* = − 0.48, *SD* = 0.53), with 20 effect sizes being negative (i.e., the higher the level of anxiety, the lower the social competence). The remaining two effect sizes were positive (i.e., the higher the level of anxiety, the higher the social competence). As per Fig. 2, the weighted mean effect-size was − 0.48 (95% *CI* = − 0.71, − 0.26), and was found to be significantly greater than zero (*z* = -4.19, *p* < .001). Therefore, across studies a significant, small, and negative relationship between anxiety and social competence in autistic children was identified.

A sensitivity analysis was conducted to ascertain any change in the estimated overall mean effect size in the context of a small (as opposed to large) level of heterogeneity. Assuming *I*^*2*^ = 25%, the estimated mean effect size was − 0.37 (95% *CI* = − 0.43, − 0.32) and was found to be significantly greater than zero (*z* = -13.17, *p* < .001). As such, a significant, small and negative relationship was still identified between anxiety and social competence in autistic children across studies, even in the context of a small amount of heterogeneity.

To determine the impact of individual studies (and therefore potential outliers) on overall pooled effect size, an influence analysis was conducted. The adjusted estimates for individual studies ranged from − 0.55 to − 0.45, with the overall estimate (-0.48) falling within the 95% confidence interval for each individual adjusted estimate. As such, no individual studies required removal.

#### Impact of Social Competence Measure on Heterogeneity of Results

Given the variability in social competence measures across the included studies, a subgroup analysis was conducted to investigate whether the strength of the relationship between anxiety and social competence in autistic children varies depending on the social competence measured. Of the 22 samples included, five used broad measures of social competence and 17 used specific, targeted measures (see Table 2). The weighted mean effect size was significantly greater than zero for samples that measured the relationship between anxiety and social competence when both broad and specific measures were used. While 95.79% of the total between-study variation was systematic in the overall meta-analysis, stratifying by social competence measure (broad/specific) indicated 77.69% systematic variance within studies using broad measures of social competence and 95.57% within studies using specific measures of social competence. The amount of systematic variance within both types of social competence measures was found to be similar (*Q*_*b*_ = 0.63, *p* = .429), meaning that the degree of heterogeneity explained did not significantly vary by social competence measure used (broad or specific).

**Table 2:** *Cochran’s Q and Stratified Test Results by Social Competence Measure Used*

	Heterogeneity					Pooled estimates		
		*Q*	*p*-value	*I* ^*2*^	*M*		*CI* (95%)	
Broad		15.30	0.004	77.69%	− 0.60		− 0.87	− 0.34
Specific		536.07	< 0.001	95.57%	− 0.45		− 0.73	− 0.17

#### Impact of Intellectual Disability (ID) Inclusion on Heterogeneity of Results

A subgroup analysis was conducted to investigate whether the degree of the relationship between anxiety and social competence in autistic children varies depending on whether the studies included children with a co-occurring ID. Of the 22 samples included, eight included children with a co-occurring ID and eight did not. The remaining six studies did not mention whether they included children in their study who had a co-occurring ID. As per Table 3, the weighted mean effect size was significantly greater than zero for studies that included children with a co-occurring ID, and for studies that explicitly mentioned they did not. While 95.79% of the total between-study variation was systematic in the overall meta-analysis, stratifying by literature type indicated 95.01% systematic variance within studies that included children with a co-occurring ID and 81.92% within studies that did not included children with a co-occurring ID. The amount of systematic variance within articles that included and did not include children with a co-occurring ID did not significantly differ (*Q*_*b*_ = 0.01, *p* = .903).

**Table 3:** *Cochran’s Q and Stratified Test Results by Inclusion of Children with a Co-Occurring ID*

	Heterogeneity					Pooled estimates		
		*Q*	*p*-value	*I* ^*2*^	*M*		*CI* (95%)	
ID Included		102.81	< 0.001	95.01%	− 0.55		− 0.86	− 0.25
ID Not Included		38.62	< 0.001	81.92%	− 0.53		− 0.78	− 0.28

### Moderator Analyses

Meta-regression analyses were conducted to investigate whether the type of social competence measure used, inclusion of children with co-occurring ID, proportion of males, age of children, and study quality moderate the relationship between anxiety and social competence in autistic children (see Table 4). The effect size of each study represents the outcome variable (such that negative effect sizes represent that the lower the levels of social competence, the higher the child’s level of anxiety). The proportion of males within a sample, age of children, and study quality were continuous variables. The type of social competence measure used had two levels (i.e., broad vs. specific), and so was dummy coded such that the reference category was the category of studies with the largest effect size (i.e., broad). Similarly, the inclusion of children with co-occurring ID had two levels (included vs. not included) and so was dummy coded such that the reference category was the category of studies with the largest effect size (i.e., ID included).

The type of social competence measure used, inclusion of children with co-occurring ID, proportion of males, and study quality did not significantly moderate the relationship between anxiety and social competence in autistic children. However, the age of the children significantly moderated this relationship, such that the older the children, the weaker the relationship between anxiety and social competence. A large amount of heterogeneity remained for all moderator variables, with the type of social competence measure used explaining 4.33% (*I*^*2*^ = 95.67) of the between-study variation, inclusion of children with co-occurring ID explaining 8.50% (*I*^*2*^ = 91.50), proportion of males explaining 6.30% (*I*^*2*^ = 93.70), age of children explaining 6.41% (*I*^*2*^ = 93.59), and study quality explaining 4.05% (*I*^*2*^ = 95.95).

**Table 4:** *Meta-Regression Test Results for Predicted Moderator Variables*

Comparison	β	*SE*_*β*_	*p*-value
Broad vs. specific measure	0.17	0.28	0.555
ID included vs. ID not included	− 0.02	0.20	0.920
Proportion of male children	0.01	0.02	0.394
Age of children	0.02	0.01	0.001
Study quality	0.02	0.07	0.787

### Publication Bias and Small Study Effects

**Fig. 3:**
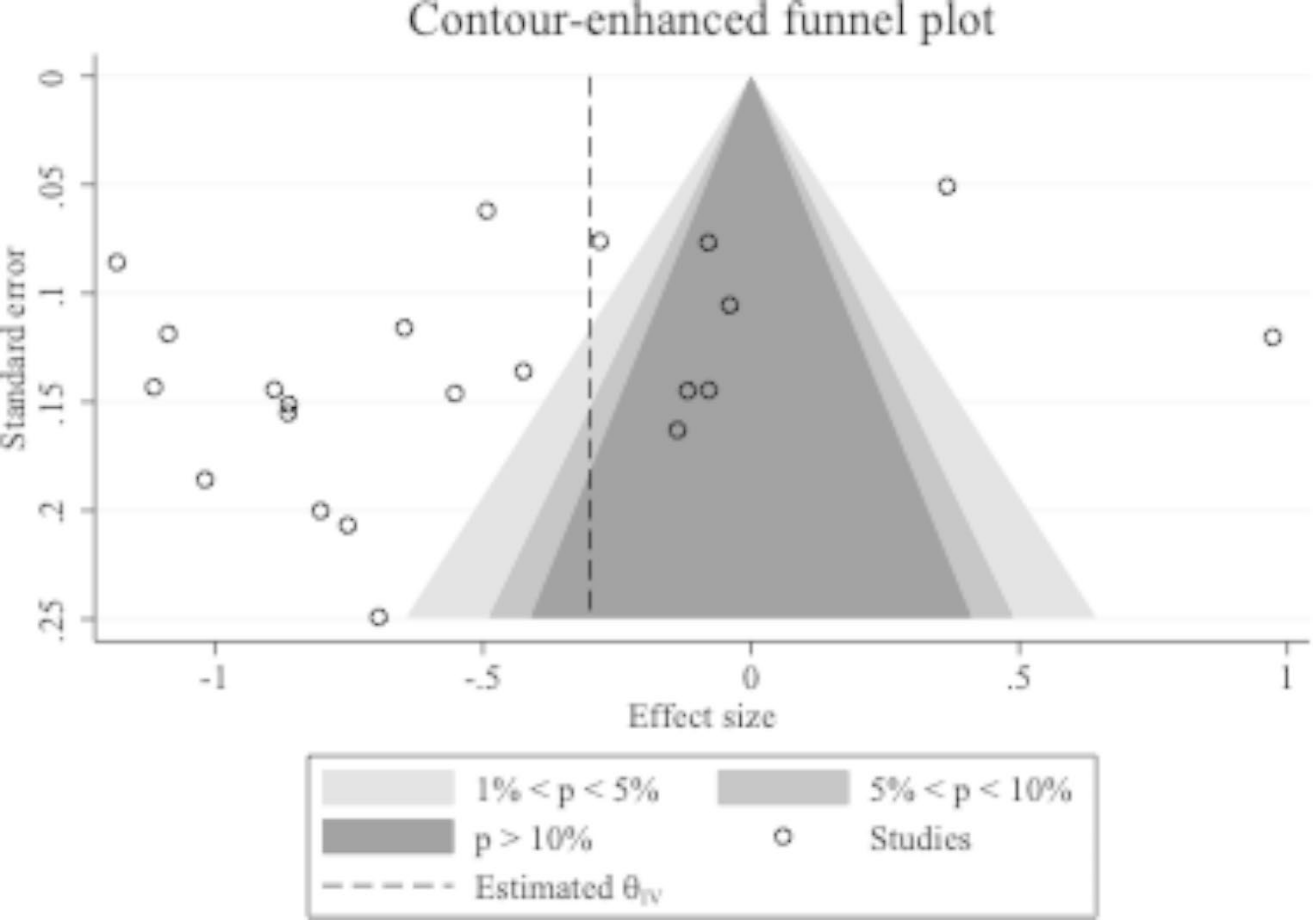
*Contour-Enhanced Funnel Plot of Included Studies*

Consistent with Rücker et al. (2011), several methods were used to assess the data for publication bias and small study effects. First, a contour-enhanced funnel plot of the effect size of each study against its standard error was visually inspected (see Fig. 3). The contour-enhanced funnel plot shows an asymmetrically distributed pattern of effect sizes, suggesting that there may be an underrepresentation of studies with positive effect sizes. As such, the strength of the overall effect size may have been overestimated due to possible publication bias.

**Fig. 4:**
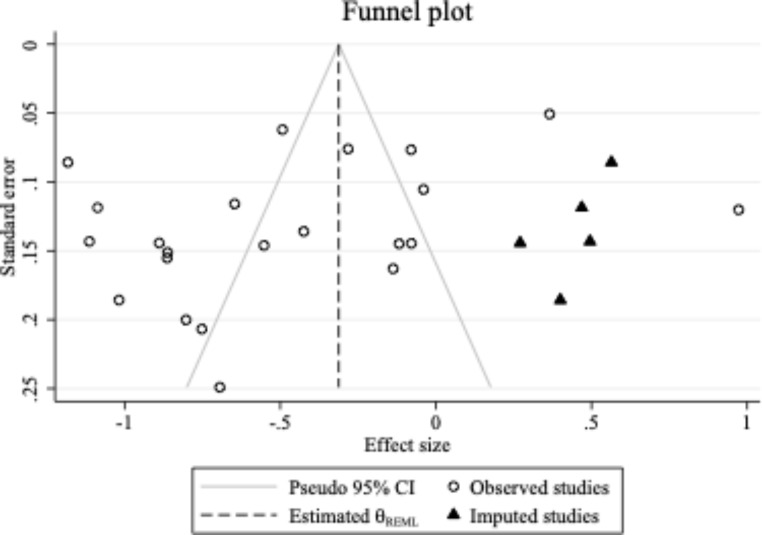
*Funnel Plot of Imputed and Included Studies*

Given the visual asymmetry of the contour-enhanced funnel plot, the trim-and-fill method (Duval & Tweedie, 2000) was used to provide an estimate of the number of missing studies, and to impute the effect sizes of any identified missing studies. Five missing studies were identified, resulting in an adjusted mean effect size of − 0.31 (95% *CI* − 0.54, − 0.08) compared to the unadjusted, original mean effect size of − 0.48 (95% *CI* − 0.71, − 0.26; see Fig. 4).

To further assess for publication bias or small study effects, Egger’s test was conducted on the overall, subgroup, and moderator analyses. Egger’s test was non-significant for the overall analysis (*p* = .100), proportion of male participants (*p* = .074), and study quality (*p* = .103). However, it was positive for inclusion of ID (*p* = .041) and age of children (*p* = .034). Overall, there was inconsistent evidence for publication bias or related small study effects in the current meta-analysis.

## Discussion

This meta-analysis examined the relationship between anxiety and social competence in autistic young people (2–18 years). By combining 22 independent samples of data from 20 studies, two key findings were evident. First, there was a significant, small, and negative relationship between anxiety and social competence in autistic young people. This suggests that those with higher anxiety levels also have lower scores on measures of social competence. However, whilst the result is significant (and remains significant even if the heterogeneity is reduced), as 95.79% heterogeneity in the scores remains unexplained, anxiety is clearly only one factor, amongst others, that is associated with social competence scores in autistic young people. Further analyses were undertaken to explore potential reasons for this large amount of heterogeneity. This led to the second key finding of the meta-analysis, which is that of the moderating and influencing factors explored, only participant age significantly moderated the relationship between anxiety and social competence, such that the negative relationship between anxiety and social competence weakens with increasing age. As these findings are discussed below, consideration must be given to the potential for publication bias which may impact the overall effect size and possible the moderating impact of age.

The negative direction of the relationship between anxiety and social competence in autistic individuals highlighted through the meta-analysis is reflective of that observed for neurotypical individuals in correlational research (e.g., Bornstein et al., 2010; De Lijster et al., 2018). Spence and Rapee’s (2016) model of the development of anxiety offers a possible explanation for the negative relation between anxiety and social competence. This model posits that adverse learning experiences may result in negative self-schemas and self-beliefs. Within a social context, for example, if a child experiences negative social interactions, they may develop negative self-beliefs which, in turn, may lead to increased levels of anxiety. As noted by Ambrose et al. (2021), there may be further catalysts into this model for autistic children, who may experience more negative social responses (e.g., social rejection, bullying) than non-autistic children (Park et al., 2020). The majority of studies in this meta-analysis utilise cross-sectional designs, which prevents any conclusions about the direction of the relationship between anxiety and social competence. However, the two longitudinal studies included in this meta-analysis conclude that social competence does not predict anxiety (Duvekot et al., 2018; Teh et al., 2017), supporting Gotham et al.’s (2015) finding that anxious/depressive symptoms in autistic children predicted social difficulties in adulthood. This therefore highlights that, although the finding that there is a negative relationship between anxiety and social competence is the same in autistic and non-autistic children, it is important to consider autism-specific pathways to which this relationship is established.

One possible autism-specific explanation that may help to elucidate the negative relationship between social competence and anxiety in autistic individuals relates to bidirectionality of communication, the double empathy problem (Milton, 2012) and the impact on neurotypical ratings of social competence. However, as none of these variables were considered in the meta-analysis, this should only be considered one of many possible explanations for the findings. Research has highlighted a mismatch between the communication styles of autistic and neurotypical individuals (e.g., Crompton et al., 2020) and thus any breakdown in communication does not lie with one interaction partner alone, but instead relates to the intersection between the two partners (Jones et al., 2021). Interview studies with autistic adults have highlighted how autistic people’s well-being is deeply affected by social misunderstandings and rejections, often leading to avoidance and withdrawal with the aim of self-perseveration (Camus et al., 2022). It is possible that autistic young people become anxious about being perceived as having poorer social competence due to ongoing feelings of being misunderstood and/or rejected. Indeed, autistic people describe experiences of acceptance and belonging as a crucial aspect of their well-being (Camm-Crosbie et al., 2019) and theoretical models posit that autistic individuals who report poorer belonging are at increased risk of going on to experience higher levels of anxiety (Shochet et al., 2016). Viewing the results through the lens of the double empathy problem allows an alternative anxiety prevention/intervention pathway to be suggested; one that places the onus upon the non-autistic community to reduce discrimination and stigma against autistic people. This could have even greater potential to improve autistic well-being and to reduce anxiety if combined with supporting young people to develop an autistic identity and social identification (e.g., Cooper et al., 2023). However, such autism-specific pathways of anxiety and social competence development require exploration in high-quality longitudinal studies.

## Moderators of the Relation between Anxiety and Social Competence

Five factors that may impact the relationship between anxiety and social competence were examined: gender, the presence of co-occurring ID, study quality, type of social competence measure, and child age. Child age was the only significant moderator, showing that, as the child’s age increases, the relationship between anxiety and social competence became weaker. One possible explanation for the relationship between anxiety and social competence reducing in adolescence compared to childhood is the change in phenomenology of anxiety with increasing age. Whilst separation anxiety is more prevalent in younger children (Wijnhoven et al., 2018), performance and social anxiety is higher in adolescent autistic children (den Houting et al., 2018). As such, differing factors may influence both the onset and maintenance of anxiety across development. In addition, the transition between childhood and early adolescence is characterised by changes in the trajectory of anxiety symptomatology and associated outcomes in non-autistic children (Xu et al., 2021). These have then been linked to a range of individual stressors and supports for anxious children (Nelemans et al., 2018) as well as to cognition (Miers et al., 2013), theory of mind (Ronchi et al., 2020), and peer victimisation experiences (Sukhawathanakul & Leadbeater, 2020). Considering how these factors may impact the relationship between anxiety and social competence in autistic children and how that relationship may change as they transition into adolescence could be a fruitful avenue for future research.

In addition to the age-related changes in anxiety that have been documented in autistic children and youth, the presentation of social competence skills may be affected by the multiple personal and environmental changes that can occur, particularly during adolescence. As these may change the perceived level of social competence, they may in turn impact the relationship between anxiety and social competence. It has been suggested that, in non-autistic youth, compensatory factors such as social supports and skills may impact the relationships between anxiety and social outcomes over time (Miers et al., 2013), so it may be that this also applies to autistic children who access supports or who use camouflaging or masking to adjust perceptions of their social competence. Mitchell et al. (2021) built the double empathy problem theory in their model to suggest that autistic people whose social behaviour is negatively interpreted by others may also be more likely to camouflage or mask their autism. Camouflaging is both driven by and linked to anxiety as well as to autism-related stigma in autistic adults (Perry et al., 2022). There have been limited studies documenting camouflaging across childhood (Cook et al., 2021), but there is some preliminary evidence that camouflaging increases from childhood into adolescence (Simcoe et al., 2018), aligning with increases in social demands (Cook et al., 2021). Adolescents who begin to mask or camouflage will score higher on measures of social competence, which may then impact the relationship between the social competence scores and anxiety. This may be one possible explanation for the finding that the relationship between anxiety and social competence is less strong in adolescents than in children, something that could be explored in future studies which include measures of camouflaging and fear of negative evaluation (see Cooper er al., 2023) alongside measures of social competence and anxiety.

Many skills and opportunities change with age which may impact the relationship between social competence and anxiety. In autistic adolescents, but not in non-autistic controls, both social anxiety and behavioural avoidance increase with age (Kuusikko et al., 2008). It is possible that adolescents have more autonomy in relation to their social environments, enabling them to avoid those in which they feel more anxious and/or less socially competent, thereby weakening the relationship between anxiety and their reported social competence. Associated with the developmental changes of adolescence, it is also possible that the factors that impact social competence become more complex for autistic individuals during this stage of life. For example, self-esteem has been implicated as a mediator between anxiety and social skills in adolescent autistic females (Jamison & Schuttler, 2015). It may therefore be that the relationship between anxiety and social competence weakens due to the increased influence of intermediary factors with increasing age; future research may explore potential mediators between anxiety and social competence.

## Limitations and Future Research

The aim of this meta-analysis was to explore the association between anxiety and social competence in autistic young people. All of the measures are measures of social competence, and predominantly based on parent report, indicating the need for more research using self-report measures of social competence which reflect autistic communication skills and preferences. Some of the measures of social competence were subscales from autism diagnostic measures, such as specific scores from the ADOS. Whilst these subscales may measure an element of social competence, it is important to acknowledge that social competence skills are not the same as autism characteristics; correlations between these areas are related but separate (e.g. O’Loghlen and Lang, 2023). Additionally, non-autistic people experience challenges in social competence (e.g. Scheerer et al., 2020).

Social competence is only one aspect of social outcomes, as categorised by de Lijster et al. (2018), and may be influenced by broader social outcomes (Joy, 2016) including social relationships, social acceptance, or victimisation. The limited availability of research precluded meta-analysis of the relationship between anxiety and these broader social outcomes, highlighting the need for further research into the relationship of anxiety to social outcomes beyond social competence in autistic children and adolescents. The measures of anxiety used in the studies within this meta-analysis were predominantly unidimensional, so the relative contributions of specific forms of anxiety (for example, social anxiety) cannot be examined; an observation which is important to consider when interpreting the meta-analysis findings. There is therefore a need for more work reporting on specific anxiety types, so that the impact of social anxiety (for example) over other types of anxiety can be better understood, as this would inform ways avenues for supports. Although over 27,500 articles were identified and then reviewed in the search, it is likely that there are other studies which included measures of anxiety and social outcomes, but as their title, abstract or keywords did not refer to social outcomes, they were not identified in the search conducted for this study.

Notably, it is possible that the individual effect sizes used within this meta-analysis may be impacted by same-rater/shared variance bias, due to the same-rater (e.g., a parent) completing questionnaires on both anxiety and social competence characteristics. This potential bias may lead to stronger (or inflated) correlations between anxiety and social competence. As such, future correlational studies exploring anxiety and social competence should aim to explore an individual’s anxiety and social competence using multiple raters.

Despite exploring five moderator variables, the large proportion of between-study heterogeneity remained unexplained in the meta-analysis (95.79%), suggesting that there are other factors that must influence the relationship between anxiety and social competence. Two possible factors are language ability and executive function skills, both of which have been associated with social skills and/or anxiety for neurotypical individuals (Hebert-Myers et al., 2006; Snyder et al., 2014). Furthermore, social competence is a complex construct that is influenced by individual and situational factors (Joy, 2016). Therefore, the heterogeneity could be explained through investigating interactions between combinations of moderator variables. To date, however, there are insufficient studies to obtain enough statistical power to examine these interactions. Further research is needed to examine other potential moderators of the relationship between anxiety and social competence.

## Conclusions

The current meta-analysis aimed to ascertain the strength, and potential moderators, of the relationship between anxiety and social competence in autistic young people. The results indicate a small, significant negative relationship between anxiety and social competence, and the strength of this relationship reduces with age. social competence, however, is complex and the current knowledge on factors influencing social competence in autistic young people is sparse. The findings of this meta-analysis suggest that further research is needed to better understand whether individual and contextual factors may impact both the trajectory of anxiety over time, and the development of social competence for autistic individuals, in order to inform supportive practices.

## Data Availability

All data search chains, tables and analyses outputs are publicly available OSF page, linked in the article.
